# AgCNER, the First Large-Scale Chinese Named Entity Recognition Dataset for Agricultural Diseases and Pests

**DOI:** 10.1038/s41597-024-03578-5

**Published:** 2024-07-12

**Authors:** Xiaochuang Yao, Xia Hao, Ruilin Liu, Lin Li, Xuchao Guo

**Affiliations:** 1 College of Land Science and Technology, China Agricultural University, Beijing, 100083 China; 2 College of Information Science and Engineering, Shandong Agricultural University, Tai’an, 271000 China; 3 College of Information and Electrical Engineering, China Agricultural University, Beijing, 100083 China

**Keywords:** Agriculture, Agroecology, Scientific data, Computational science

## Abstract

Named entity recognition is a fundamental subtask for knowledge graph construction and question-answering in the agricultural diseases and pests field. Although several works have been done, the scarcity of the Chinese annotated dataset has restricted the development of agricultural diseases and pests named entity recognition(ADP-NER). To address the issues, a large-scale corpus for the Chinese ADP-NER task named AgCNER was first annotated. It mainly contains 13 categories, 206,992 entities, and 66,553 samples with 3,909,293 characters. Compared with other datasets, AgCNER maintains the best performance in terms of the number of categories, entities, samples, and characters. Moreover, this is the first publicly available corpus for the agricultural field. In addition, the agricultural language model AgBERT is also fine-tuned and released. Finally, the comprehensive experimental results showed that BiLSTM-CRF achieved F_1_-score of 93.58%, which would be further improved to 94.14% using BERT. The analysis from multiple aspects has verified the rationality of AgCNER and the effectiveness of AgBERT. The annotated corpus and fine-tuned language model are publicly available at https://doi.org/XXX and https://github.com/guojson/AgCNER.git.

## Background & Summary

As a basic sub-task of information extraction, named entity recognition (NER) plays an important role in many natural language processing tasks such as relation extraction, question answering, etc.^1^, aiming to extract the named entities from the unstructured texts. Over the years, scholars worldwide have conducted in-depth NER research in various languages such as Chinese^2^, English^3^, and Arabic^4^, as well as in various vertical fields such as social media^5^, biomedicine^6^, and cybersecurity^7^. In recent years, NER has gradually been applied to the field of agricultural diseases and pests, used to identify the entities related to agricultural diseases and pests such as “小麦 (wheat)” and “玉米大斑病 (Corn northern leaf blight)”. It is an important component of downstream tasks such as agricultural knowledge graph construction and intelligent question answering, and has important practical significance and theoretical research value for improving the efficiency and accuracy of crop diseases and pests diagnosis and control, reducing food losses, and ensuring food security^8^.

In recent years, various deep learning-based NER methods have been proposed. According to the main types of neural networks, it can be divided into various classic NER models based on CNN-CRF;^9,10^, BiLSTM-CRF;^11,12^, and Transformer^13–15^. Moreover, according to the proposed chronological order, it can be divided into traditional statistical machine learning methods^16^, traditional word embedding-based models^12^, external feature enhancement models;^15,17–19^, and large-scale pre-trained language models;^20,21^. In addition, various NER tools such as NLTK^22^ and spacy^23^ have also been constructed. However, the pre-trained language model oriented to agricultural diseases and pests has not been reported. Nevertheless, the ADP-NER task is still challenging due to the scarcity of publicly available datasets, which is the core foundation for this domain research. Therefore, it is necessary to establish a comprehensive and unified benchmark corpus to fill the gap in ADP-NER tasks.

To achieve this goal, many efforts that attempt to construct agriculture-related NER corpora have been made as detailed in Table 1. Malarkodi *et al*.^24^ constructed the first agricultural-oriented dataset by using CRF, which included 19 fine-grained tags and 11,041 entities. Bisbas *et al*.^25^ introduced WordNet to construct an agricultural dataset and dictionary that includes 5 major categories, i.e., *grains*, *fruits*, *nuts*, *spices*, and *vegetables*. However, they mainly focus on English and are not applicable to Chinese, let alone agricultural diseases and pests. To this end, Li *et al*.^26^ collected a dataset for crop diseases and pests, which contains 3 entity categories, i.e., *crops*, *pests and diseases*, and *pesticides*. Zhang *et al*.^27^ further divided a new type, i.e., *fertilizer*. Finally, a dataset containing 4 entity categories was constructed.

**Table 1:** The details of the existing agricultural named entity corpora.

Works	Year	Language	Entity Tags	No. of Entities	No. of Instances	No. of Tokens	Objects	Available
Malarkodi *et al*.^24^	2016	English	19	11,041	—	100,000	Agriculture	✗
Biswas *et al*.^25^	2016	English	5	—	—	171,735	Agriculture	✗
Li *et al*.^26^	2017	Chinese	3	—	74,429	—	Agriculture	✗
Zhang *et al*.^27^	2018	Chinese	4	11,108	114,002	—	Agriculture	✗
Qian *et al*.^28^	2023	Chinese	6	8,873	—	—	Agriculture	✗
Wei *et al*.^29^	2022	Chinese	4	29,790	37,243	1,800,000	Agriculture	✗
Jiang *et al*.^30^	2023	Chinese	9	48,000	—	—	Agriculture	✗
Chen *et al*.^31^	2019	Chinese	16	150,000	—	—	Agriculture	✗
Shen *et al*.^32^	2020	Chinese	4	7,380	1,581	—	Rice	✗
Zhang *et al*.^33^	2021	Chinese	21	11,876	5,574	130,448	Apple	✗
Liu *et al*.^34^	2022	Chinese	16	11,670	6,000	22,000	Wheat	✗
Yan L^35^	2021	Chinese	10	17,671	7,828	430,000	Grape	✗
Guo X^37^	2020	Chinese	11	350,725	34,952	420,000	Agricultrue	✗

To solve the problems of the limited and low quality of the agricultural dataset, Qian *et al*.^28^ implemented a BiLSTM-CRF model. However, their dataset only contains 6 categories and 8,873 entities, which is relatively small in scale. Wei *et al*.^29^ focused on the issues of insufficient extraction in terms of character position, context, and long-distance dependency, and constructed an agricultural dataset containing 37,243 samples and 29,790 agricultural entities. However, it only includes 4 main types. Moreover, Jiang *et al*.^30^ regarded the authoritative agricultural books as the data sources, and introduced active learning and crowdsourcing to build a dataset containing 9 types of entities and more than 48,000 entities. But it has not been made public. Besides, Chen *et al*.^31^ constructed a relatively complete agricultural knowledge graph AgriKG, which defined 16 entity categories such as *animal*, *plant*, and *agricultural products*, etc., including more than 150,000 entities, and realized the entity retrieval and question answering.

In addition, some works mainly revolved around only one crop. Shen *et al*.^32^ labeled 1581 samples of rice diseases and pests based on 4 types of entities such as *disease*, *pest*, *weeds*, and *pesticide*, and achieved better recognition results on the proposed JE-DPW model. In addition, Zhang *et al*.^33^ divided the apple-related entities into 21 categories and constructed a dataset ApdCNER for Chinese apple named entity recognition. The F_1_-score of 92.14% on ApdCNER was implemented by using the novel proposed APD-CA model. Moreover, several works also annotated the NER datasets for specific crops such as wheat^34^ and grapes^35^, but the limitation is that they cannot effectively cover other crops^36^. More importantly, as marked by ✗, all the corpora mentioned above are not publicly available, which undoubtedly hinders the ADP-NER task.

In summary, efforts have been made in the past to collect and annotate agricultural datasets. Nevertheless, constructing such an ADP-NER-oriented benchmark dataset faces two main challenges. (1) There is no publicly available dataset for the ADP-NER task. To achieve the ADP-NER tasks, it is necessary to first self-construct a dataset, which will be time-consuming. (2) Although the above works can provide some reference for the construction of the corpus in entity categories division, there are still shortcomings in terms of entity categories, sample size, and crop coverage. To tackle the abovementioned issues, Based on existing works;^8,37^, we constructed a large-scale dataset named AgCNER for the Chinese named entity recognition in the agricultural diseases and pests domain. It contains a total of 206,992 entities and 66,553 samples, with over 3 million characters, including 13 categories such as Crop, Disease, Pest, Drug, Cultivar, Fertilizer, Company, *et al*. The original texts come from the Internet and have a certain degree of diversity and universality. In addition, a series of NER models on AgCNER were trained, and the pre-trained language model for ADP-NER tasks named AgBERT was also fine-tuned to dynamically generate high-quality contextual semantic representations with rich domain information. Finally, The domain dataset AgCNER and fine-tuned language model AgBERT are also made publicly available on GitHub, which is the first time in the agricultural diseases and pests field, even in the agriculture domain.

## Methods

### Entity types

Most general domain-oriented datasets focus on annotating the common entity types such as Person, Location, and Organization, while the vertical domain-oriented datasets such as Biomedical^38^, Archaeological^39^, and Geographic Information^40^ focus on the domain entity types such as 慢性阻塞性肺疾病(chronic obstructive pulmonary disease), 斧头(Axe), and 通济渠(Tongji Canal). However, due to the differences across domains, the aforementioned domain entities do not apply to the agricultural diseases and pests field. Considering that there is currently no unified standard for entity types division, this paper divided the entities of agricultural diseases and pests into 10 entity types, including *Crop*, *Disease*, *Pest*, *Drug*, *Fertilizer*, *Pathogens*, *Period*, *Part*, *Cultivar*, and *Biosystematic* according to the previous agricultural works;^24,28,30–32^,. In addition, several general entity types such as *Organization* and *Company* were also considered. Moreover, the category *Other* was also introduced to mark the potential entities. Finally, 13 categories were divided and their specific details were shown in Table 2.

**Table 2:** Details of entity categories in AgCNER.

#	Tags(CN)	Tags(EN)	Abbr.	Examples
1	虫害	Pest	PET	棉铃虫(Bollworm), 甜菜夜蛾(Spodoptera exigua)
2	病害	Disease	DIS	赤霉病(Fusarium head blight), 稻瘟病(Rice blast disease)
3	作物	Crop	CRO	水稻(Rice), 苹果(Apple), 黄瓜(Cucumber)
4	药剂	Drug	DRUG	敌敌畏(Dichlorvos), 多效唑(Paclobutrazol)
5	品种	Cultivar	CUL	九丰7号(Jiufeng-7), 吉粳801(Jijing-801)
6	肥料	Fertilizer	FER	氮肥(Nitrogen fertilizer), 磷肥(Phosphate fertilizer)
7	病原	Pathogens	PAOG	大豆疫霉菌(Phytophthora sojae), 稻瘟菌(Magnaporthe grisea)
8	周期	Period	PER	扬花期((Flower period), 抽穗期(Heading date)
9	部位	Part	PART	叶(Leaf), 芽鞘(Coleoptile), 前翅(Fore wing), 大脑(Brain)
10	公司	Company	COM	长沙长龙生物科技有限公司(Changsha Changlong Biotechnology Co., LTD), 浙江农科种业有限公司(Zhejiang agricultural science seed industry Co., LTD)
11	组织机构	Organization	ORG	山东农业大学(Shandong Agricultural University), 中国水稻研究所(China National Rice Research Institute)
12	生物介元	Biosystematic	BIS	同翅目(Homoptera), 粉虱科(Aleyrodidae)
13	其他	Other	OTH	低毒杀虫剂(Low toxicity insecticide), 病毒性病害(Viral disease)

### Data acquisition

To obtain sufficient corpus, seven of the most authoritative data sources related to agricultural diseases and pests have been considered as listed in Table 3. Among them, China National Knowledge Infrastructure, Wangfang Data, and Baidu Baike were selected to crawl the abstracts of the articles or semi-structured data by using the specific diseases or pests (https://github.com/guojson/AgCNER/blob/main/diseases_and_pests) as keywords such as “小麦赤霉病(Wheat scab)” and “稻瘟病(Rice blast)”. The irrelevant texts were removed under the guidance of experts. Moreover, to ensure diversity, we also obtained various related texts from multiple platforms such as the China Agricultural Technology Promotion Information Platform and China Pesticide Information Network. The domain experts were also invited to verify to ensure the relevance and accuracy of the samples. After the preprocessing such as deduplication and denoising, we obtained 66,553 original samples.

**Table 3:** The details of data sources for the dataset annotated by authors.

#	Data source	URL
1	China National Knowledge Infrastructure	https://www.cnki.com.cn/
2	Wangfang Data	https://www.wanfangdata.com.cn/
3	China Agricultural Technology Promotion Information Platform	https://njtg.nercita.org.cn/user/index.shtml
4	China Pesticide Information Network	http://www.chinapesticide.org.cn/
5	Agricultural Diseases, Pests, and Weeds Image and Text Database	http://bcch.ahnw.cn/
6	Chinese Crop Germplasm Information System	https://www.cgris.net/disease/default.html
7	Baidu Baike	https://baike.baidu.com/

### Annotation tool and process

**Fig. 1:**
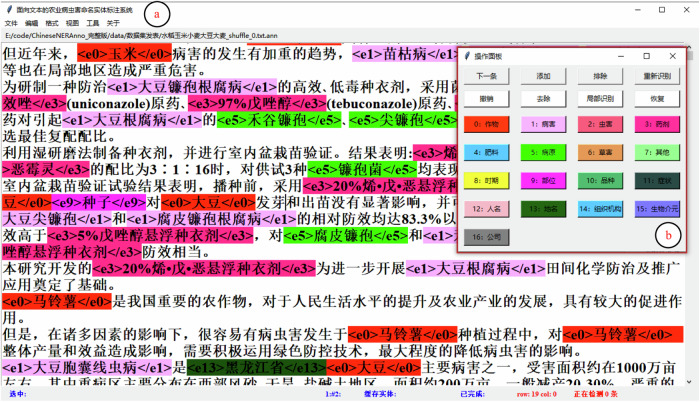
The main userface of ChineseNERAnno.

**Fig. 2:**
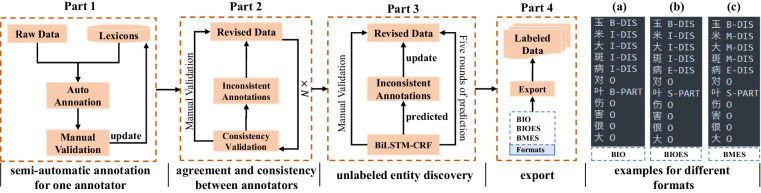
Main workflow for annotating AGCNER dataset.

To improve annotation efficiency, a new named entity annotation tool named ChineseNERAnno (https://github.com/guojson/ChineseNERAnno.git) has been developed in our previous work^37^. It is a general NER annotation tool that is not only applicable to Chinese NER tasks but also to other languages with spaces as separators, such as English, Arabic, German, etc. It’s front-end mainly includes the main interface (a) and operation panel (b) as shown in Fig. 1. Among them, the operation panel pre-defined the buttons with different entity categories to mark the entities in text with corresponding categories. The main interface was used to visualize the samples to be labeled, and entities were displayed with different formats “ < e1 > < /e1 > ” and colors according to their respective category. The category numbers were aligned with category names as shown in Table 2. For example, given an entity belonging to *Disease* can be labeled with the mark pairs “ < e2 > < /e2 > ”. In addition, to ensure the consistency and accuracy of annotation, a data dictionary was built based on the SQLite database, which was conducive to realizing semi-automatic through entity matching. Moreover, the data dictionary can be updated by continuously incorporating new entities. Besides, to avoid confusion with the original data, the annotated corpus will be saved as a text file with the suffix “.ann”, and its format is shown in Fig. 1. The final corpus will be converted into text files with suffix “.txt” through “导出(Export)” menu according to different label formats, i.e., BIO (B-begin, I-inside, O-outside) as shown in Fig. 2(a), BMES (B-begin, M-middle, E-end, S-single) as shown in Fig. 2(b), or BIOES (B-begin, I-inside, O-outside, E-end, S-single) as shown in Fig. 2(c).

To ensure the quality of the dataset, and improve the speed and consistency of annotation, the corpus was annotated by 3 annotators under the guidance of domain experts. The basic process of data annotation is shown in Fig. 2, which was generally divided into four parts, i.e., semi-automatic annotation, agreement and consistency between annotators, unlabelled entity discovery, and export.

It is worth noting that both auto annotation and rigorously checked annotation are considered throughout the annotation process. One common sense is that automatic annotation will show poor performance at the beginning of annotation because of the limited annotated entities in the dictionary. Therefore, it relies more on manual annotation to expand the vocabulary and correct incorrectly annotated entities. However, the advantages of automatic annotation will become increasingly prominent with an increasing number of annotated entities expanded into vocabulary. Meanwhile, manual validation is also required to discover the entities with incorrect annotations and discover new entities.

Besides, considering that different annotators may have different understandings of the entity types, resulting in annotation conflicts. Therefore, the strict manual verification with the correction principle, i.e., minority obeys majority, is conducted to correct annotation errors and unify the annotators’ cognition after labeling a certain number of samples. Meanwhile, Fleiss’ Kappa^41^, donated as ***k***, was used in the inter-rater agreement. The larger the ***k***, the more consistent the annotated results. It is generally believed that when ***k*** > 0.8, there is almost perfect consistency among annotators.

1 $$k=\frac{{p}_{o}-{p}_{e}}{1-{p}_{e}}$$

2 $${p}_{o}=\frac{1}{N}\mathop{\sum }\limits_{i=1}^{N}\frac{1}{n(n-1)}[\left(\mathop{\sum }\limits_{j=1}^{k}{n}_{{ij}}^{2}\right)-n]$$

3 $${p}_{e}=\mathop{\sum }\limits_{j=1}^{m}{\left(\frac{1}{{Nn}}\mathop{\sum }\limits_{i=1}^{N}{N}_{{ij}}\right)}^{2}$$

where *n*, *N*, and *m* represent the number of annotators, characters, and entity types respectively. *p*_*e*_ is the sum of squares of the joint edge distributions for each entity type *j*; *P*_*o*_ is the average of the percentage of consistent annotation pairs for each type among all annotation pairs.

### Quality control

In summary, we adopt various strategies for quality control. In the data acquisition stage, several authoritative and standardized domain portals were also selected as the main data source. Meanwhile, some samples from Baidu Baike were also collected to improve the diversity of the corpus. In the data processing stage, besides the data cleaning and noise removal, correlation screening was also conducted under the guidance of domain experts. In the data annotation stage, multiple measures such as annotation tool, semi-automatic recognition, manual validation, agreement and consistency between annotators, and model prediction were also considered to alleviate annotation conflicts while improving annotation speed. Moreover, the BiLSTM-CRF model was introduced to discover unlabelled entities as much as possible. More detailed guidelines about the annotation process can be found on the figshare platform at 10.6084/m9.figshare.c.6807873.v1^42^.

## Data Records

According to the statistics, we corrected approximately 121,700 entities during manual annotation, including 98,748 real entities and 22,952 pseudo entities with incorrect labels. Moreover, with an increase in the annotated samples, the frequency of manual validation also decreased gradually, thus accelerating the annotation speed.

**Fig. 3:**
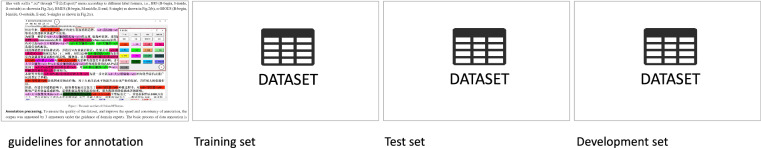
Overview of the datasets publicly available on the figshare platform.

**Fig. 4:**
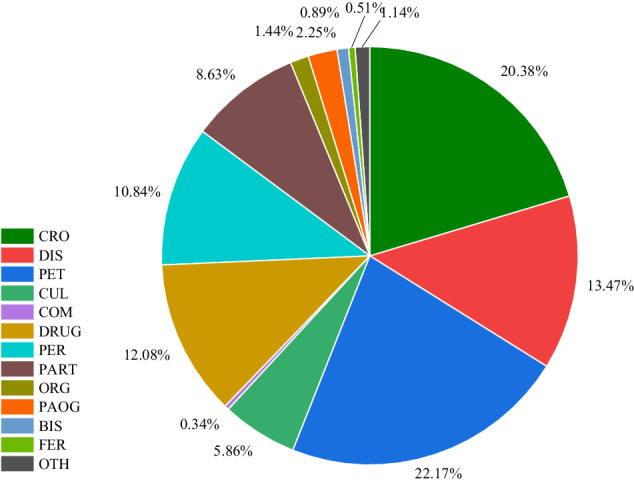
Quantity distribution for each category in AgCNER.

Finally, a large-scale Chinese named entity recognition corpus for agricultural diseases and pests named AgCNER was constructed. For the convenience of users and to evaluate the effectiveness of the novel ADP-NER models, the annotated corpus is publicly available on the figshare platform at 10.6084/m9.figshare.c.6807873.v1^42^. It contains a total of 13 categories, 66,552 annotated samples, 206,992 entities, and 3,909,293 characters. As shown in Fig. 3, AgCNER mainly contains four files, i.e., a training set with the text file named ‘train.txt’ that contains 47840 sentences, a development set with the text file named ‘dev.txt’ that contains 5964 sentences, a test set with the file named ‘test.txt’ that contains 5975 sentences according to a ratio of 8:1:1 and the format of BIO, and the guidelines about the annotation tool and process. As shown in Fig. 2a,each character is stored on a single line with a corresponding BIO label in the text file and separated by a space. Several examples are shown in Table 4. The statistical analysis of the distribution of each type of entity in AgCNER is shown in Fig. 4. It can be seen from the figure that several types such as *CRO*, *PET*, and *DIS* account for a relatively large proportion, indicating that they maintain a higher level of attention and will be easier to be identified. However, the proportion of several entities such as *COM*, *BIS*, and *FER* is relatively low, leading to lower predicted F_1_ values.

**Table 4:** Several samples of named entity recognition of Chinese crop diseases and pests.

Sentence	稻粒黑粉病主要侵染时期在水稻齐穗至灌浆初期;每公顷用18.7%的灭黑灵450g防效最高, 保产率可达18.12%。
Sentence (English)	The main infection periods of Rice Kernel Smut are from full heading to the early filling stage of rice; The highest control effect can be achieved by using 450 g of 18.7% Diniconazole per hectare, with a yield of 18.12%.
Labels	B-DIS,I-DIS,I-DIS,I-DIS,I-DIS,O,O,O,O,O,O,O,B-CRO,I-CRO,B-PER,I-PER,O,B-PER,I-PER,I-PER, I-PER,O,O,O,O,O,O,O,O,O,O,O,B-DRUG,I-DRUG,I-DRUG,O,O,O,O,O,O,O,O,O,O,O,O,O,O,O,O,O,O,O,O,O
Entities	病害: 稻粒黑粉病; 作物: 水稻; 时期: 齐穗, 灌浆初期; 药剂: 灭黑灵
Entities (English)	DIS: Rice Kernel Smut; CRO: Rice; Period: full heading, early filling stage; DRUG: 18.7% Diniconazole
Sentence	水稻是我国的主要粮食作物, 有甬籼57、新两优6号、中浙优1号等品种, 发生较普遍且危害严重的病虫害主要有稻白叶枯病、稻瘟病、三化螟, 褐飞虱、白背飞虱和稻飞虱等。其中, 稻白叶枯病是由水稻黄单胞杆菌侵染引起的细菌病害, 可采用50%氯溴异氰尿酸、20%噻菌铜进行防治。
Sentence (English)	Rice is one of the main grain crops in China, with many varieties such as Yongxian-57, Xinliangyou-6, and Zhongzheyou-1. Generally, there are many serious diseases and pests against rice such as Rice bacterial leaf blight, Rice blast, Tryporyza incertulas, Nilaparvata lugens, Sogatella furcifera, and Rice planthopper. Among them, the Rice bacterial leaf blight is a bacterial disease caused by the Xanthomonas oryzae, which can be controlled with 50% chlorobromoisocyanuric acid and 20% thiamethoxazole.
Labels	B-DIS,I-DIS,O,O,O,O,O,O,O,O,O,O,O,O,B-CUL,I-CUL,I-CUL,I-CUL,O,B-CUL,I-CUL,I-CUL,I-CUL, I-CUL,O,B-CUL,I-CUL,I-CUL,I-CUL,I-CUL,O,O,O,O,O,O,O,O,O,O,O,O,O,O,O,O,O,O,O,O,O,B-DIS,I-DIS,I-DIS,I-DIS,I-DIS,O,B-DIS,I-DIS,I-DIS,O,B-DIS,I-DIS,I-DIS,O,B-DIS,I-DIS,I-DIS,O,B-DIS,I-DIS,I-DIS,I-DIS,O,B-DIS,I-DIS,I-DIS,O,O,O,O,B-DIS,I-DIS,I-DIS,I-DIS,I-DIS,O,O,B-PAOG,I-PAOG,I-PAOG,I-PAOG,I-PAOG,I-PAOG,I-PAOG,O,O,O,O,O,O,O,O,O,O,O,O,O,B-DRUG,I-DRUG,I-DRUG,I-DRUG,I-DRUG,I-DRUG,I-DRUG,I-DRUG,I-DRUG,O,B-DRUG,I-DRUG,I-DRUG,I-DRUG,I-DRUG,I-DRUG,O,O,O,O,O
Entities	病害: 稻白叶枯病、稻瘟病；作物: 水稻；病原: 水稻黄单胞杆菌；药剂: 50%氯溴异氰尿酸、20%噻菌铜；虫害: 三化螟、褐飞虱、白背飞虱、稻飞虱；品种: 甬籼57、新两优6号、中浙优1号
Entities (English)	DIS: Rice bacterial leaf blight, Rice blast; CRO: Rice; PAOG: Xanthomonas oryzae; DRUG: 50% chlorobromoisocyanuric acid, 20% thiamethoxazole; PET: Tryporyza incertulas, Nilaparvata lugens, Sogatella furcifera, Rice planthopper; CLU: Yongxian-57, Xinliangyou-6, and Zhongzheyou-1

In addition, we also compared AgCNER with some other agricultural corpora listed in Table 1. Most datasets such as Biswas *et al*.^25^, Li *et al*.^26^, and Zhang *et al*.^27^ only contain several typical categories such as *DIS*, *PET*, *DRUG*, etc. The categories *SYMPTOM* and *PART* have been pre-designed by Chen *et al*.^31^ and Zhang *et al*.^33^, but they are not publicly available. Compared to our previous work^37^, two new categories,i.e., *ORG* and *COM* were further annotated, and the number of categories was ultimately increased to 13. Besides, AgCNER contains 206,992 entities, which exceeds most datasets listed in Table 1 except^37^. Still, our corpus has a larger scale with 66,553 samples and 3,909,293 characters, indicating richer semantic information and is more practical.

## Technical Validation

In this section, a variety of mainstream NER models were selected for various experiments and discussions to promote the widespread use of AgCNER and provide a reference for future NER research in agricultural diseases and pests field. The deep learning framework was Pytorch 1.12.1 based on NVIDIA GeForce RTX 3090 GPU and i9-12900K CPU.

### Evaluation metrics

In this paper, three commonly used metrics, i.e., Precision(P), Recall(R), and F_1_-score(F_1_) were selected as the evaluation indicators. The entity is considered to be correctly predicted only when its boundaries and tag are correctly recognized.

4 $$P{recision}=\frac{{TP}}{{TP}+{FP}}\times 100 \% $$

5 $$R{ecall}=\frac{{TP}}{{TP}+{FN}}\times 100 \% $$

6 $${F}_{1}=\frac{2\times P\times R}{P+R}\times 100 \% $$

Among them, *TP* represents the number of samples that correctly predicted positives concerning the ground truth labels, *FP* means the number of samples that predicted positive but negative ground truth labels, *FN* represents the number of samples that predicted negative but positive ground truth labels.

### Comparison models

To comprehensively evaluate the quality of the constructed dataset, various representative baselines were introduced, which can be roughly divided into four categories,i.e., traditional machine learning models such as HMM and CRF, word embedding-based models such as BiLSTM-CRF, IDCNN-CRF, pre-trained language model-based methods such as BERT-CRF、BERT-BiLSTM-CRF、BERT-IDCNN-CRF, and external feature enhancement-based models such as Lattice-LSTM^18^, TENER^15^, Flat-Lattice(FLAT)^43^, NFLAT^44^, HNER^45^. BiLSTM-CRF is the earliest recognized classic NER model based on deep learning that utilizes the bidirectional LSTM to extract contextual semantic features and CRF as the decoder. In addition, the domain PLM named AgBERT was also obtained by fine-tuning the original BERT based on the agricultural corpus, and its effectiveness was discussed.

### Hyper-parameters setting

For BiLSTM-CRF and IDCNN-CRF, the *embedding size* was set to 128, *batch size* to 32, to alleviate the over-fitting problem, *dropout* was introduced and set to 0.6. We selected Adam as the optimizer. The learning rate (*lr*) was set to 1e-3. To improve the training performance, *lr* would be adjusted during the training process by an effective decay strategy StepLR with the multiplier *gamma* 0.8 and interval *step_size* 5. The *epoch* was set to 30 with *patience number* 5, i.e., if the F_1_ value on the development dataset does not obtain a higher value within 5 epochs, the training process would be terminated. For IDCNN-CRF, *kernel size* was set to 3 with the dilation 1, and *the number of filters* was 128. For BiLSTM, *the hidden size* of BiLSTM was set to 384. For the PLM-based models, the *batch size* was set to 50. Moreover, AdamW was selected as the optimizer with the ReduceLROnPlateau method to improve the fine-tuning performance. The learning rates of PLM, BiLSTM, IDCNN, Linear, and CRF were set to 3e-5, 1e-3, 1e-3, 1e-3, and 1e-3 respectively. It is worth noting that when PLM is not fine-tuned, the parameters of PLM will be frozen.

### Quality control

**Fig. 5:**
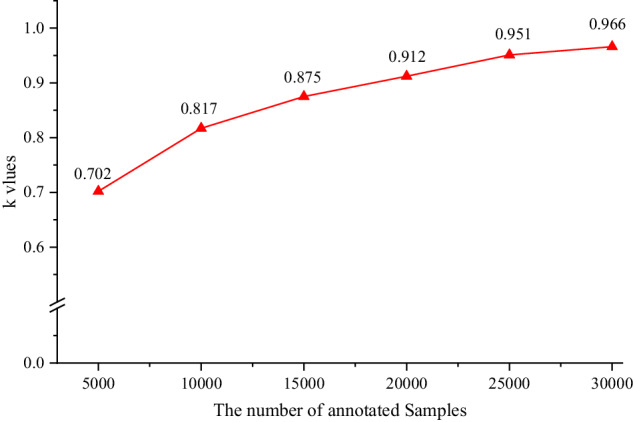
Experimental results about consistency evaluation.

As seen from the previous context, Fleiss’ Kappa was considered for annotation consistency evaluation in this paper. Every 5000 annotated samples were evaluated. According to Fig. 5, a low ***k*** value was obtained when a total of 5000 samples were annotated in the first round for the reason that annotators have different understandings of domain entities and annotation rules at this stage, resulting in significant differences in annotation. Moreover, it gradually increased with the number of annotated samples increases. The ***k*** value slowed down when the total number of the annotated samples reached 30000, indicating that the cognitive unity among annotators was gradually reached. Meanwhile, ***k*** > 0.8, it could be considered that the annotated dataset is credible.

**Fig. 6:**
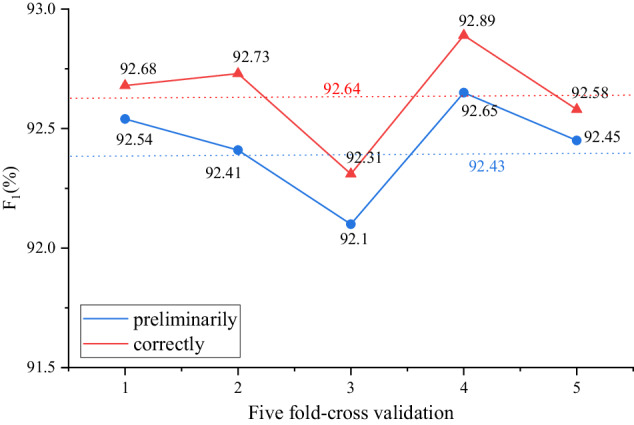
The five-fold-cross validation.

To discover potential unlabelled and incorrectly labeled entities in the preliminarily annotated dataset, BiLSTM-CRF was conducted to 5-fold cross-validation. The F_1_ values after validation and correction are shown in Fig. 6. The gaps were relatively lower in each fold, and the average F_1_ value of the preliminary dataset is 92.43%. According to statistics, a total of about 560 new entities were discovered, mainly focusing on the categories *PART* and *OTH*. Because they have limitations such as diverse expression forms and blurred boundaries, which increases the difficulty of labeling. However, after the correction, the average F_1_ value increased by 0.21 percentage points, reaching 92.64%, indicating the effectiveness of the five-fold cross-validation.

### Division Evaluation for AgCNER Dataset

**Fig. 7:**
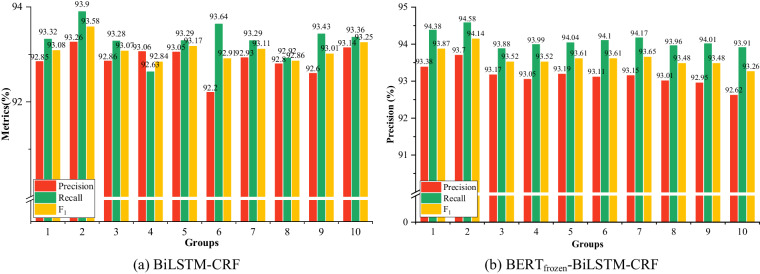
The macro results of BiLSTM-CRF and BERT-BiLSTM-CRF on AgCNER.

Reasonable dataset partitioning is a prerequisite for training NER models, which may contribute to ensuring the efficient utilization of data and improving the generalization of the NER models. In this section, the ten-fold cross-validation was introduced, i.e., first, the whole dataset was divided into an average of 10 parts. Then, two of them were randomly selected as the development set and test set respectively, while the rest would be merged and regarded as the training set. We selected the basic mode BiLSTM-CRF and BERT-BiLSTM-CRF with frozen weights, denoted as BERT_frozen_-BiLSTM-CRF as benchmark models. The results are shown in Fig. 7 and Table 5. Figure 7 shows the macro results of benchmark models on AgCNER, while Table 5 lists the detailed results of baselines for each type of category.

**Table 5:** The detailed results of the baselines for each type of entity.

Models	Metrics	BIS	OTH	COM	CRO	DIS	DRUG	FER	ORG	PART	PER	PET	PAOG	CUL
BiLSTM-CRF	Precision	91.38	64.51	71.42	95.28	97.86	90.41	85.57	70.99	88.96	89.79	96.65	91.57	88.45
	Recall	93.92	52.83	81.89	96.05	97.81	90.74	86.86	73.88	88.83	90.68	96.99	93.21	90.04
	F_1_	92.62	57.69	75.93	95.66	97.83	90.57	86.16	72.37	88.89	90.23	96.82	92.35	89.23
BERT_frozen_-BiLSTM-CRF	Precision	91.11	62.01	71.49	96.06	97.57	91.66	85.36	71.92	90.00	90.49	96.77	87.69	89.02
	Recall	96.69	58.25	83.38	96.28	97.94	92.56	90.41	80.76	90.77	91.31	97.24	91.79	91.30
	F_1_	93.81	60.03	76.72	96.17	97.75	92.11	87.76	76.07	90.38	90.90	97.00	89.65	90.14

We can find in Fig. 7 that different results would be obtained on the datasets merged with different partitions. Both BiLSTM-CRF and BERT_frozen_-BiLSTM-CRF obtained the highest F_1_ scores of 93.58% and 94.14% respectively in 2_nd_ group. Therefore, we selected the 2_nd_ group as the optimal dataset. Moreover, BERT_frozen_-BiLSTM-CRF achieved an average F_1_-score of 93.61%, which is 0.3% higher than BiLSTM-CRF, indicating the intuitive common tense that BERT_frozen_-BiLSTM-CRF on tan datasets were generally higher than those of BiLSTM-CRF.

In Table 5, BiLSTM-CRF and BERT_frozen_-BiLSTM-CRF both achieved excellent results on *DIS*, *PET*, and *CRO*, which demonstrated their easy recognition because of the relatively clear boundaries and sufficient quantity. For example, the entities belonging to *DIS* such as ‘小麦纹枯病(Wheat Sharp Eyespot)’ and the entities belonging to *PET* such as ‘大豆食心虫(Soybean Pod Borer)’ usually contain the fixed word, i.e., ‘病(disease)’ and ‘虫(pest)’, which would be regarded as the delimiters that contribute the entity detection. However, some types of entities such as *ORG*, *COM*, and *OTH* were difficult to recognize because of the boundary ambiguity and complex composition. Overall, the experimental results demonstrated the effectiveness of the corpus, and the optimal dataset could be regarded as a benchmark dataset for the named entity recognition task in the agricultural diseases and pests domain.

### Main results

The experimental results for all models on the dataset annotated by the authors were visualized in Table 6. Among them, BERT_frozen_ represents the original BERT that does not participate in fine-tuning during the training process, while AgBERT indicates the agricultural BERT fine-tuned on the agricultural corpus. Compared to other models, HMM performed the worst because it is an independent assumption model, which would be limited in capturing complex contextual information and label dependencies. Different from HMM, CRF significantly improved the performance of NER for the reason that it can consider global information and learn more contextual relationships. In addition, the label transition probability matrix also enhances the ability to handle complex dependency relations between labels.

**Table 6:** Experimental results of all models on AgCNER dataset.

Model	Deep Learning	External Features	LLM	Precision	Recall	F_1_
HMM	✗	✗	✗	63.58	72.02	67.54
CRF	✗	✗	✗	94.16	92.2	93.17
BiLSTM-CRF	✓	✗	✗	93.26	93.9	93.58
BiLSTM-Attention-CRF	✓	✗	✗	94.04	93.04	93.53
IDCNN-CRF	✓	✗	✗	92.38	93.37	92.88
Lattice-LSTM	✓	Character-word Lattice	✗	90.4	93.13	91.74
TENER	✓	Character and Word-level Embeddings	✗	92.79	94.95	93.85
FLAT	✓	Flat Lattice	✗	93.65	94.87	94.26
NFLAT	✓	Lexicon	✗	**94.21**	**95.10**	**94.66**
Graph4CNER^48^	✓	Lexicon, Collaborative Graph Network	✗	92.84	93.59	93.22
BERT_frozen_-BiLSTM-CRF	✓	✗	✓	93.70	94.58	94.14
BERT_frozen_-IDCNN-CRF	✓	✗		93.26	93.10	93.18
AgBERT-BiLSTM-CRF	✓	✗	✓	94.01	94.68	94.34
AgBERT-IDCNN-CRF	✓	✗	✓	93.55	94.39	93.97
HNER	✓	Subword sequence	✓	93.76	93.92	93.84

Moreover, with the introduction of BiLSTM for feature extraction, BiLSTM-CRF has improved the F_1_-score by 0.41%. Its advantages lie in two aspects. (1)There is no need for manual feature engineering, which will alleviate human error. (2) The global sequence context features can be effectively modeled. Furthermore, BERT-based BiLSTM-CRF achieved better performance than word embedding-based models. Specifically, BERT_frozen_-based BiLSTM-CRF and IDCNN-CRF increased their F_1_-values by 0.56 and 0.3 percentage points compared to word2vec-based ones, indicating that the character-level semantic representation generated by original BERT is richer than that generated by word2vec. Moreover, under the fine-tuning, i.e., AgBERT, the F_1_ values were further increased by 0.2% and 0.79%, reaching 94.34%, and 93.97% compared with BERT_frozen_-based BiLSTM-CRF and IDCNN-CRF respectively, indicating the effectiveness of AgBERT that fine-tuning contributes BERT to learning domain knowledge and then generating high-quality text representations.

TENER achieved a higher F_1_ value of 93.85% than the above-mentioned models other than AgBERT-based ones, for the reason that it proposed a novel attention that incorporates the direction and relative distance of the words, which are both important for the NER tasks. Moreover, except for the word-level information, the character-level information in a word was also considered. Lattice-LSTM contains a character-word lattice structure that can dynamically match the lattice and incorporate the word information. However, it obtained a relatively lower F_1_ value on the AgCNER dataset, i.e., 91.74%, due to the agricultural text and insufficient ability to capture long-distance dependencies. To remedy the deficiencies of low inference speed, FLAT was proposed to convert the lattice structure into flat span structure^43^. The experimental results listed in Table 6 showed that FLAT exhibited excellent parallelization ability and its F_1_ increased by 2.52% compared to Lattice-LSTM. NFLAT further improved the F_1_ to 94.66% by proposing InterFormer, a novel lexical enhancement method that contains non-flat lattices, which can reduce the amount of computational and memory costs. By comparing the experimental results mentioned above, it was found that models such as TENER, FLAT, NFLAT, and HNER are generally superior to CRF-based models. This is because existing research has shown that external features such as Lexicon, Flat Lattice, Subword sequence, character level, or word level features are conducive to enriching contextual semantic information and improving the recognition accuracy of NER models. It also showed that they were suitable for the dataset annotated by the authors.

### Detailed results for each category

To further evaluate the predicted results from a microscopic perspective, five classical models, i.e., BiLSTM-CRF, IDCNN-CRF, BERT_frozen_-BiLSTM-CRF, AgBERT-BiLSTM-CRF, and HNER were selected and corresponding experimental results for each category were detailed in Table 7. Among them, the optimal results on each evaluation metric for each entity category were highlighted in Bold format and different colors, i.e., red indicates the optimal P, green indicates the optimal R, and black indicates the optimal F_1_. All benchmark models generally achieved high F_1_ values for several categories such as *CRO*, *DIS*, *DRUG*, and *PET*. Taking *DIS* as an example, its highest F_1_ was 97.99% produced by AgBERT-BiLSTM-CRF, while the lowest one was still as high as 97.59%. On the contrary, the models performed not well in some categories such as *OTH* and *ORG*. For example, the lowest F_1_-value of 53.33% for *OTH* was achieved by IDCNN-CRF, while the highest one was only 59.76% obtained by AgBERT-BiLSTM-CRF, expressing more obvious difficulty-to-recognition. The reasons may include the following two aspects: (1) limited domain corpus leads to insufficient training. For example, *ORG* listed in Fig. 4 only accounts for the overall 0.34%. (2) The complexity of the composition of the domain-named entities such as *OTH*, often consists of multiple types of characters such as Chinese characters, numbers, and characters, which can easily interfere with recognition results to a certain extent. Therefore, it is extremely challenging to identify the aforementioned entities. In addition, AgBERT-based BiLSTM-CRF achieved optimal F_1_ and recall values in most categories such as *OTH*, *CRO*, *DIS*, and *CUL*, demonstrating the effectiveness of BERT fine-tuning in detail. In the future, more advanced models will be proposed to improve the recognition accuracy of agricultural diseases and pests named entities.

**Table 7:** Detailed results for each category on AgCNER.

### Agricultural pre-trained language modals

**Fig. 8:**
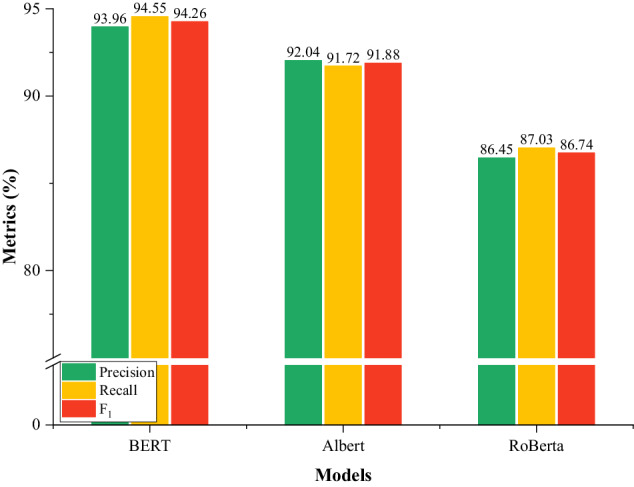
Experimental results of CRF with different PLMs.

In recent years, large-scale pre-training language models (PLMs) such as BERT have been widely used in many NLP tasks because of their strong semantic representation ability. Meanwhile, the experimental results in Table 5 and Table 6 also indicate the effectiveness of PLMs to improve the performance of named entity recognition. However, there is no publicly available PLM in agricultural field. In this section, the domain PLM, named AgBERT, was obtained by fine-tuning the original BERT on the agricultural corpus. To demonstrate the rationality of selecting BERT, the experiments were conducted by selecting CRF as the decoder and introducing other two PLMs, i.e., Albert and RoBerta as the comparison model. As shown in Fig. 8, BERT-CRF outperformed other models and achieved the best F_1_ value of 94.26%. In addition, the experimental results in Table 6 indicate that BERT-BiLSTM-CRF also achieved higher F_1_ than BERT-CRF under the fine-tuning condition, indicating that the character-level semantic representation generated by BERT may be more conducive to improving the performance of NER models.

**Fig. 9:**
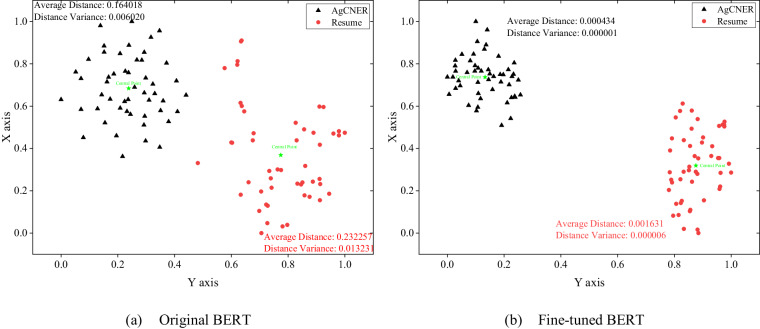
The visualization of the sentence-level semantic representations on AgCNER and Resume.

To further confirm the above inference, this section took BERT as an example and randomly selected 50 sentences from the AgCNER and Resume datasets respectively, and their sentence-level semantic representations were generated by using the pre-trained language models mentioned above. Then, the average semantic representation was obtained according to the sentence length. In this paper, T-SNE^46^ was introduced to reduce the 768 dimensions into 2 dimensions to visualize the semantic representation by using the rectangular coordinate system. As shown in Fig. 9, the closer the two points separated from the space, the more similar the sentence semantics, and the greater the possibility of belonging to the same field. Common sense is that data points belonging to the same dataset should be spatially distributed in one cluster. Compared to the original BERT, the fine-tuned BERT, i.e., AgBERT not only shortened the average distance (0.000434 for AgBERT and 0.164018 for original BERT on AgCNER) and the distance variance (0.000001 for AgBERT and 0.006020 for original BERT on AgCNER) between the inner-cluster nodes and central node (marked by ⋆) but also divided all points that belong to the same domain into their corresponding clusters with obvious boundaries, indicating that the nodes generated by AgBERT are more similar and demonstrating the effectiveness and superiority of the fine-tuned BERT in representing the domain characters. Therefore, the above experimental results not only demonstrated the rationality of selecting BERT, but also reveal the effectiveness of AgBERT after fine-tuning on domain corpus.

### Error analysis

**Fig. 10:**
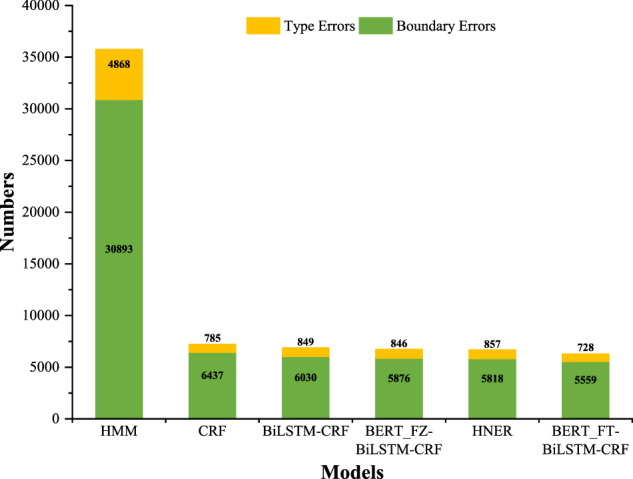
Error statistics for the baselines on AgCNER.

**Fig. 11:**
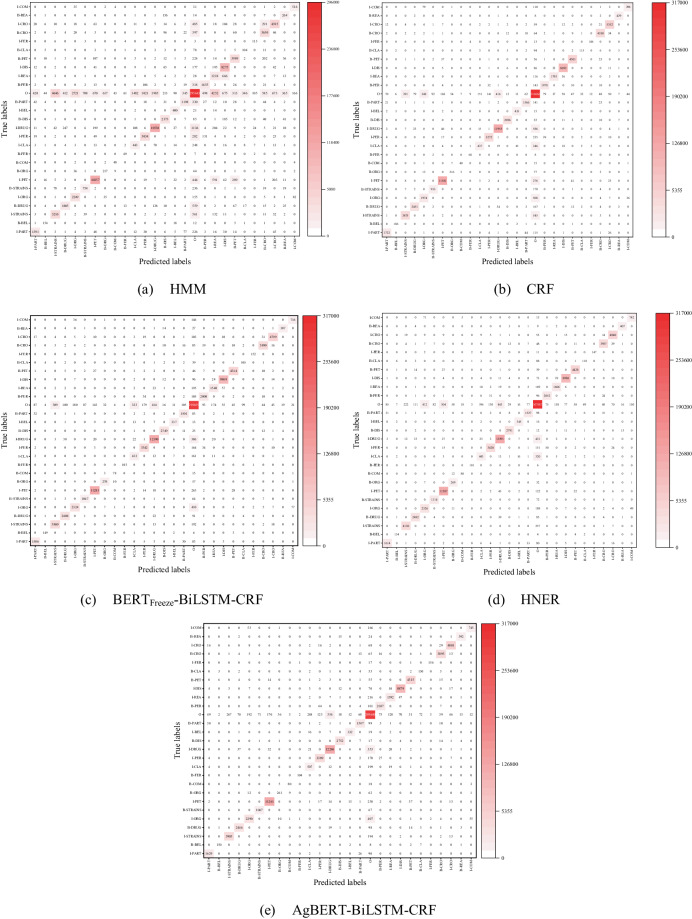
The visualization of the sentence-level semantic representations on AgCNER and Resume.

Considering the possible errors in manual annotation, we took the results predicted by HMM, CRF, BERT_Freeze_-BiLSTM-CRF(BERT-FZ-BiLSTM-CRF), AgBERT-BiLSTM-CRF, and HNER as an example to analyze the possible errors that may occur during the prediction and annotation process. According to^47^, we divided the possible types of errors into boundary errors and entity-type errors. As shown in Fig. 10, boundary errors are the leading cause of the final predicted errors, while entity-type errors are the secondary cause. In contrast, the pre-trained model can alleviate such problems to some extent due to its strong domain representation ability. To report a detailed analysis of the possible errors, the confusion matrix on the test dataset of AgCNER was also plotted in Fig. 11. we observed that the majority of the predicted errors were caused by boundary errors. For example, *B-ORG* was mislabeled with *O* and vice versa. Therefore, the gap between the NER models and humans indicates the challenge of the dataset annotated by the authors and the importance of the domain NER tasks.

## Data Availability

All codes and fine-tuned language model AgBERT with outputs were publicly available at: https://github.com/guojson/AgCNER.git.
